# Using computed tomography, photogrammetry & structured light scanning for measuring human mandibles: a comparison of metric analysis using various 3D imaging technologies

**DOI:** 10.1007/s00414-026-03933-8

**Published:** 2026-07-28

**Authors:** Isabella Crebert, Xanthe Weston, Dilan Seckiner, Matthew Farrow, Lars C. Ebert

**Affiliations:** 1 Office of the School of Law & Justice, University of Newcastle, Newcastle, Australia; 2 Queensland Centre for Domestic and Family Violence Research, Central Queensland University, Mackay, QLD Australia; 3 University of New South Wales, Sydney, Australia; 4 School of Biomedical Sciences & Pharmacy, University of Newcastle, Newcastle, Australia; 5 Forensic Institute Zurich, Zurich, Switzerland

**Keywords:** Advanced scanning technologies, Mandible, Computed tomography, Photogrammetry, Structured light scanning, Forensic anthropology, Metric assessment, Measurement

## Abstract

This paper aims to determine if photogrammetry and/or structured light scanning (SLS) can be used as alternatives for conducting metric assessments on mandibles as opposed to computed tomography (CT). Identifying alternative imaging options to CT is important as, although CT is accurate and reliable, there are multiple limitations that impact its accessibility, including cost, portability, and ease of learning. Photogrammetry and SLS are two handheld, lightweight, cost-effective alternatives that are relatively user friendly. Therefore, this paper imaged 33 human mandibles using CT, photogrammetry, and SLS. For intra-observer error, the ICC results were between 0.37 and 1, indicating a range of unreliable to excellent measurements. Almost all TEM results were within the acceptable threshold set for this study (< 2). Despite graphical demonstrations of the measurements being provided to the observers, all inter-observer results were unreliable, indicating that further refinement of these definitions is required before a protocol can be developed. However, all inter-modality assessments were within the acceptable threshold (< 5 TEM). The range for the ICC results for CT v photogrammetry was between 0.37 and 0.88. For CT v SLS, the results were between 0.47 and 0.87. Overall, photogrammetry had the highest degree of consistency with CT, but all methods proved to be interchangeable. This paper highlights that alternative imaging options are available in scenarios where CT cannot be accessed.

## Highlights

- Both photogrammetry and SLS can produce a similar level of accuracy to CT.
- Photogrammetry achieved the highest degree of consistency compared to CT.
- The intra-observer error ranged from 0.37 to 1 ICC and 0.26–2.43 for TEM.
- All inter-observer scores were above the acceptable TEM threshold (< 2).
- All inter-modality tests were within the acceptable TEM threshold (< 5).

## Introduction

Estimating biological sex in forensic anthropology is of paramount importance, as subsequent steps, including stature and age at death estimation, are dependent on sex [1]. The mandible is a reliable indicator for biological sex estimation, especially in the absence of the pelvis [2] and cranium [3]. The mandible is the most robust bone that is likely to withstand taphonomic change [4], consequently it is often recovered intact. On average, a male mandible tends to possess more robust and pronounced features, as well as being larger in terms of length, breadth, and height, whereas the female mandible is more gracile [5]. However, there is, of course, human variation within these generalities. There are two common approaches for assessing and estimating biological sex – metric and morphological [6]. Metric analysis is concerned with the measurements of bones and how these measurements can be used to estimate biological sex. Langley et al., (2016) [7] developed the *Data Collection Procedures for Forensic Skeletal Material *(DCP), which sets out the standard measurements that should be collected on various bones, including the mandible. This protocol was developed from the results of the Langley et al.’s (2016) study which conducted 99 measurements on a sample of 50 skeletons [8]. The collection of measurements also includes mandibular measurements that are designed to be applied to physical bone. However, at the time of writing, these measurements have not been replicated on 3D models of mandibles.

The use of three-dimensional (3D) technology has presented multiple benefits to forensic anthropology that have permitted dynamic advancements in the discipline [9]. Technologies such as computed tomography (CT) offer an alternative approach to documenting and assessing skeletal remains. Utilising 3D technologies permits virtual storage and sharing of skeletal remains, both of which can be important for court purposes, particularly in the event of a re-trial where skeletal evidence may be buried. Further, the virtual storage option also allows for the creation of digital repositories or ‘virtual labs’ [9]. Having access to virtual labs helps to mitigate issues regarding access to skeletal remains for training purposes and also helps to inform secular and population changes across populations, which is vital to modern forensic knowledge, as a result of population admixture [10]. This is especially important as access to contemporary skeletal collections is limited in multiple areas, such as Australia [11] and Europe [12].

Further, whilst identification via DNA analysis is a popular and commonly utilised in forensic contexts, in the absence of being able to acquire a DNA sample, potentially as a result of not having access to a sufficient database sample, skeletal identification via a biological profile is an alternative approach [13]. Although skeletal analysis is unable to provide the identity of an individual, it does helps to narrow the pool of potential matches. This is especially important in the current political climate with (at the time of writing) more than 130 active global conflicts and the resultant prediction for an increase in demand of timely identification. Utilising 3D technology provides the opportunity for scientists to assess data in real time from any location, which may help to reduce the workload of responders and help to increase the speed of the identification process [10]. These are important considerations in contexts where there are a significant number of skeletal remains to assess, such as mass grave scenarios.

Presently, CT is considered the ‘gold standard’ for virtual skeletal analysis [2, 14] due to its high level of accuracy and reliability in terms of replicating physical bones. CT also allows for segmentation of tissue from bone, and comparison of ante and post-mortem data such as dental information. Multiple studies have validated the use CT for achieving high measurement accuracy when compared to the gross bone on the pelvis, achieving a biological sex classification accuracy range of between 62 and 100% [14–23]. One study by Jerković et al., (2022) [10] applied the cranial DCP measurements to dry crania and CT (multi-slice) 3D models and found that the method is interchangeable for these measurements, with some exceptions, between the gross and 3D bone. However, the use of CT in forensic anthropology has multiple practical limitations, including its high cost (for both traditional and mobile CT), lack of mobility (for traditional CT), and the level of expertise required [14]. Further, access to CT machines generally can be limited, particularly in post-conflict or natural disaster scenarios [24].

These limitations highlight the need for alternative 3D technologies that are cost effective and portable to be tested to determine the accuracy and reliability compared to CT. The literature highlights two alternatives, photogrammetry and structured light scanning (SLS), as these technologies yield the same or similar accuracy in terms of reconstructing bone, as well as being more cost-effective, lightweight, hand-held, and easily transportable. The advent of 3D technology presents the opportunity to digitally assess skeletal human remains, including the ability to digitally measure bones which can be used to create biological profiles. Therefore, it is imperative that measurements obtained for a virtual biological profile are accurate. However, there is a scarcity of literature regarding the accuracy of photogrammetry and SLS in terms of accurately replicating the measurements of bones, particularly the mandible. While previous research has compared the use of CT to physical bone [25], photogrammetry to physical bone [26], and CT to SLS [27], to the best of our knowledge, a study which compares the accuracy of measurements achieved across CT, photogrammetry, and SLS has not been conducted. The literature that has compared these technologies demonstrates that, overall, SLS and photogrammetry can produce a higher degree of accuracy for measurements than CT [27, 28].

Therefore, the purpose of this paper is to determine if CT, photogrammetry, or SLS is best for future 3D metric analysis and digital preservation of human remains. This paper will compare virtual measurements obtained on 3D CT, photogrammetry, and SLS models of human mandibles. The following research questions will be addressed:

- Can the standard mandibular osteometric measurements required for measuring physical mandibles be replicated on virtual 3D models?
- Can measurements taken on photogrammetric- and SLS-rendered models produce the same or similar accuracy to CT?

This paper will also identify any issues encountered by the observers when utilising the software and measuring procedure. The results of this paper will help to indicate if photogrammetry and/or SLS can produce metric results as accurately as CT. This paper will also provide commentary on the usability and accessibility requirements for the equipment necessary to complete this study. This information is important as it will help to indicate any potential challenges that may be encountered so that these can be addressed and mitigated for future research and in-field use. It is important to note that the intention of this paper is not to suggest that CT be replaced, but to recommend alternatives in scenarios where CT is not accessible.

## Materials and methods

One hundred mandibles were imaged for this study using three imaging techniques – CT, photogrammetry, and structured light scanning. Each bone was imaged by each modality, resulting in three models per bone. The mandibles used for this study are from a cold case skeletal collection housed at the Forensic Institute of Zurich, Switzerland. The acquired bones have not been identified via DNA analysis. As the focus of this paper is on the accuracy of measurements across the modalities rather than using the measurements for biological sex estimation, this sample is ideal for this project. Although the bones were in various states of preservation, the mandibles that were fragmented, particularly on the coronoid processes, were excluded from the study. Although, typically, edentulous bones are excluded from studies as edentulism often means that the sexually dimorphic characteristics have been inhibited, as this study is not concerned with identifying biological sex the one edentulous bone present in the sample was included. In total, 67 bones were excluded due to fragmentation, this resulted in a sample size of 33 mandibles, with a total of 99 3D models being created. Each mandible was scanned and the subsequent models were created using the following technologies and softwares:

### Technologies used

- Siemens Somatoms Definition Flash:

For CT, the mandibles were imaged in sets of nine (9) with a slice thickness of 0.6 mm. Artec Studio 20 (AS 20) (Luxemburg) was used to individually separate the mandibles from the scan via the segmenting tool. The 3D models were created using 3D Slicer (Boston, Massachusetts).

- GOM Atos Q (Carl Zeiss GOM Metrology GmbH, Braunschweig, Germany):

The ATOS Q structured light scanner (Carl Zeiss GOM Metrology GmbH, Braunschweig, Germany) imaged the bones at a resolution of around 100 μm [6]. This scanner projects a light pattern onto the surface, and two cameras record the distortion of the pattern to calculate the surface’s 3D topology. Structured light scanners generally provide higher resolution 3D models compared to CT and photogrammetry, though, like CT, they do not capture color. However, color can be added to the 3D models using photographs. Because scans were performed on a rotating table, bones were scanned in two phases—front and back—and these two parts were semi-automatically registered using the manufacturer’s software, removing the table semi-automatically. The resulting models were created using Zeiss Inspect (Braunschweig, Germany) and exported in STL format.

- Custom built photogrammetric scanner (Forensic Institute of Medicine, Zurich, Switzerland).

The mandibles were scanned on a custom-made photogrammetric scanner consisting of a rotating glass plate and six cameras (Canon EOS RP with a 24–105 mm lens). Scale bars with machine-readable markers were placed next to the bones as reference and for automatic scaling of the resulting models. Photos were made every 15° leading to a total of 144 photos per scan. Photogrammetric calculation of the 3D models was conducted using Reality Scan (Epic Games, Inc. Cary, NC, USA). 3D data was exported in obj format with textures.

### Measurements

Metric analysis was selected rather than geometric morphometric analysis as metric techniques are the most commonly employed and utilised for court purposes. The *Standard for Data Collection from Human Skeletal Remains* [29] and *Data Collection Protocols for Forensic Skeletal Material 2.0* [7] highlight that a mandibulometer is required for three of the standard mandibular osteometric measurements - mandibular length, maximum ramus height, and mandibular angle. Ideally, the ground truth measurements for comparing measurements would be collected on the physical bone. Although, a mandibulometer may not also be accessible, particularly due to its cost [30]. As literature highlights, CT rendered 3D models can produce a measurement accuracy of between 1 and 2 mm when compared to gross bone [15], the measurements collected on the CT models were considered to be the ground truth [31]. The measurements set out in the DCP [7] were utilised for this study. These measurements ( consist of chin height, height of the mandibular body, bigonial breadth, bicondylar breadth, minimum ramus breadth, mandibular length, and maximum ramus height. . Measuring the gonial angle and breadth of the mandibular body was not possible in the software used, therefore, these measurements were excluded. Definitions and figures for these landmarks can be found in the DCP [7]. The virtual models were all measured on Artec Studio 20 (AS 20). Various software was tested, including CloudCompare (France) and MeshLab (University of Pisa, Italy). However, AS 20 allowed for CT, SLS, and photogrammetry^1^ models to be easily measured in the one program.

**Fig. 1:**
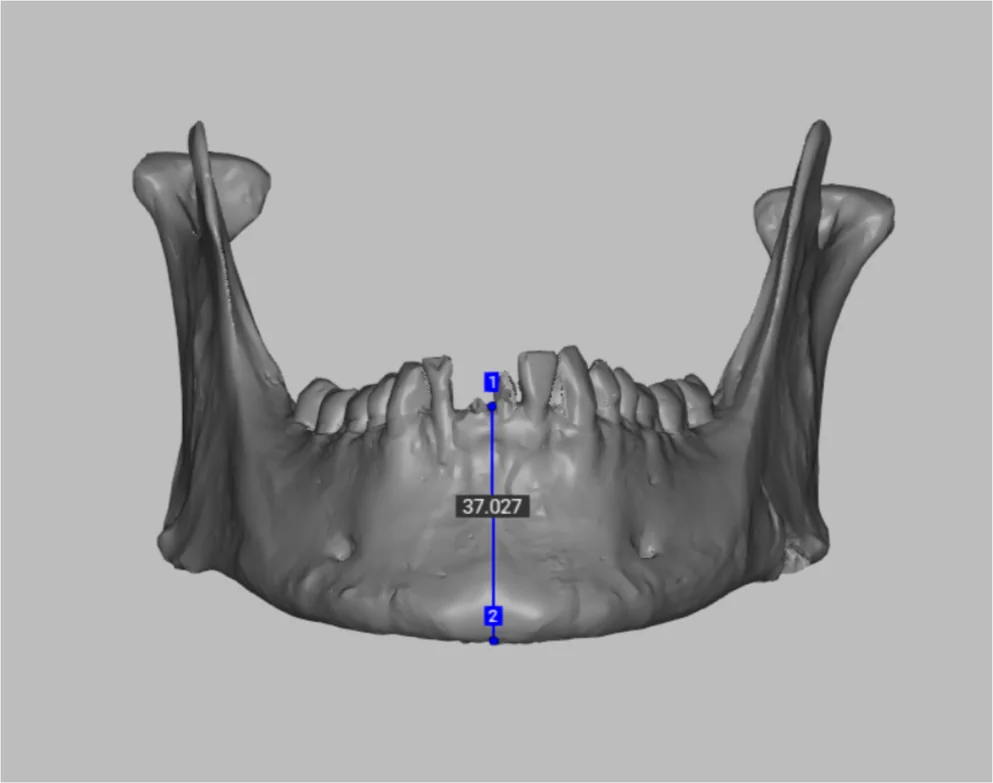
Chin height measurement in AS 20

**Fig. 2:**
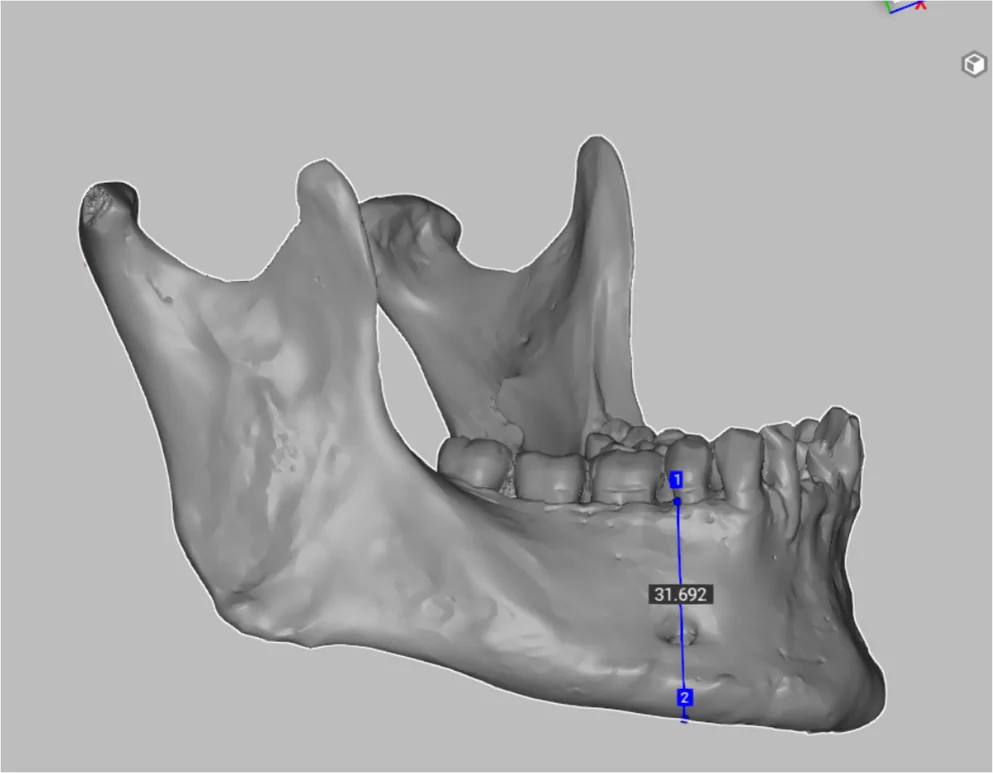
Mandibular height measurement in AS 20

**Fig. 3:**
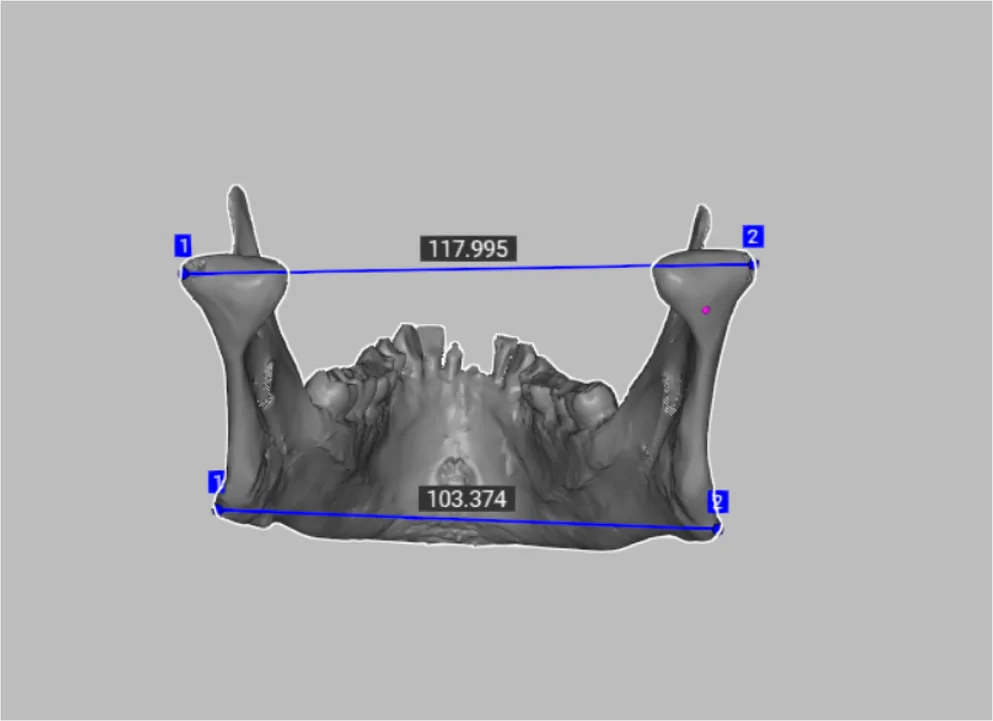
Bicondylar breadth and bigonial breadth in AS 20

The measurements are collected in AS 20 by using the linear measurement function. The observer was required to select two points on the 3D model and the measuring tool calculates the distance between in millimetres. To guide the observers on their decision for where to place the points for the measurements, they were each provided with a free digital copy of the DCP, as well as screenshots of how to replicate these measurements in a virtual environment, see Figs. 1, 2 and 3. The landmarks for the measurements are demonstrated in Table 1. Definitions and landmarks provided as per Martin & Knussman (1988) [7, 32].

**Table 1:** Points 1 and 2 to be selected on virtual 3D model of mandible for each measurement as per Martin & Knussman (1988) [7, 32]

Measurement	Point 1	Point 2	Reference image
Chin Height	Infradentale (located at the midline of the lower central incisors)	Gnathion (the most anterior-inferior point on the contour of the mandible)	Figure 1
Mandibular Body Height	Alveolar process	Inferior border of mandible in line with the mental foramen	Figure 2
Bigonial Breadth	Right gonion	Left gonion	Figure 3
Bicondylar Breadth	Most lateral point on right condyle	Most lateral point on left condyle	Figure 3
Minimum Ramus Breadth	Anterior margin of the most narrowest portion of the ramus	Posterior margin of the most narrow portion of the ramus	Figure 4
Maximum Ramus Height	Gonion	Highest part of the mandibular condyle	Figure 4
Mandibular Length	Anterior margin of the chin	Posterior border of mandibular angle	Figure 4

**Fig. 4:**
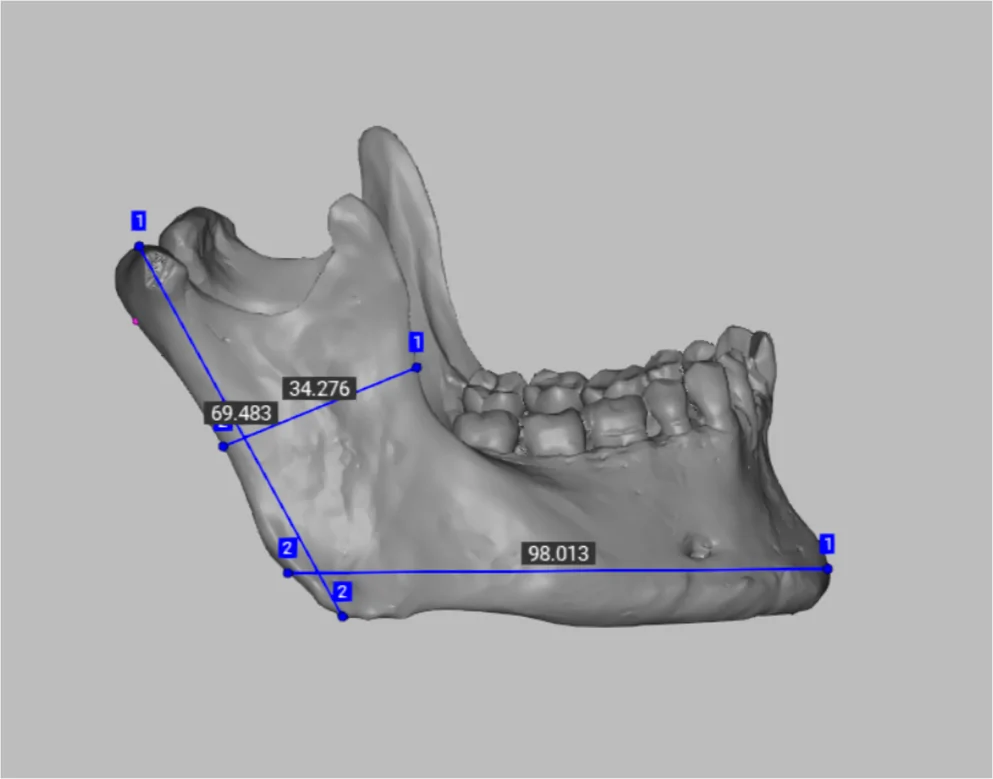
Measurements of ramus height, minimum ramus breadth, and mandibular length in AS 20

The CT rendered mandible models were considered to be the reference standard, as the measurements on the physical mandible could not be obtained. Photogrammetry and SLS derived models were isotropically scaled using CT derived chin height measurements to correct for global size differences. Models can be scaled in AS 20 using the scale feature in the tools bar. The chin height was derived from the mean of measurements collected during both observation rounds from assessor one, using the formula:

$$\:Chin\:Height\:M1\:+\:Chin\:Height\:M2\:\div\:2$$

Intra-observer reliability of chin height measurements was assessed using a two-way random effects intraclass correlation coefficient (ICC) (2,1). Reliability was excellent (ICC = 0.904; 95% CI: 0.810–0.953). The results indicate that the chin height measurement on the sample was robust and the mean of the two scores obtained by assessor one could be used to scale the photogrammetry and SLS models.

The mean was provided to all assessors to ensure that the models were consistently and accurately scaled during observation. The scale was calculated using the following equation:

$$\:R\:=\frac{x\:CT}{H\:unscaled}$$

It should be noted that the assessors did not receive this exact equation as they were blind to the fact that the models were being scaled against CT. Using the ‘scale’ function in AS, the assessors then input their resulting score to scale the models. Chin height was not included in subsequent accuracy analyses as the inclusion of this measurement would have introduced circularity and artificial inflation of agreement. All other linear mandible measurements were compared across modalities to assess measurement accuracy.

### Data analysis

Five observers measured the models to quantify the inter- and intra-observer error. The first observer measured all specimens across all modalities on two separate occasions over a period of two weeks. Five observers were provided with a random subset of (*n* = 15) of the mandibles imaged by photogrammetry and SLS (the same bones were provided for each modality). Three observers are biomedical scientists and the remaining two are forensic anthropologists, meaning that they each have a thorough understanding of human anatomy. The observers were allocated numbers based on experience with osteology, as per Fancourt & Stephen (2018). Observers 1 and 2 have a similar level of experience as forensic anthropologists (four years or more), Observers 3, 4, and 5 also have a similar level of experience as biomedical scientists (three years or more). Observer 1 has the most experience (four years), and Observer 2 is experienced in using 3D technology. Observers 3, 4, and 5 had recently undergone extensive training in using a previous version of AS within the last 12 months, at the time of writing, which was supervised by Observer 1. With the exception of Observer 1, this was the first time that the observers had measured bones in a virtual environment. Please refer to Table 2 for context on the occupation and level of experience for each observer. The varying levels of knowledge and experience help to indicate the usability of the software. Measurements were repeated twice with a two-week interval period to quantify intra-observer error and reduce recall bias, which also permitted assessment of true measurement repeatability across imaging modalities.

The observers were provided with a secure Google drive, which contained the instructions and relevant information required to complete the task, including pictures of measurement examples collected from screenshots collected in AS 20. Observers were also provided a spreadsheet to collect measurements, and de-identified 3D mandibular models presented as STLs. The STL format was selected as this means there is no colour or surface information present on the scans, just the geometric information. This was done to ensure a fair comparison was made between the modalities, as SLS and photogrammetry can produce colour and surface information, where CT cannot.

**Table 2:** Description of reviewer occupation and level of experience

Reviewer	Occupation	Level of experience with osteology	Level of experience with 3D technology
O1	PhD student	Expert	Expert
O2	Forensic anthropologist (PhD student)	Expert	Experienced
O3	Biomedical scientist	Experienced	Experienced
O4	Biomedical scientist	Experienced	Intermediate (less than one year)
O5	Biomedical scientist	Experienced	Intermediate (less than one year)

### Intra- and inter-observer

To assess the observations made by the individual observers, the first and second observation rounds were compared using the two-way, mixed effects ICC in JASP (Version 0.95.4.0). As per Koo & Li’s 2016 revision of the ICC guidelines, the interpretation is as follows:

- Excellent agreement = 0.90 or above.
- Good agreement = 0.75–0.90.
- Moderate agreement = 0.50 or above.
- Poor agreement = 0.50 or below.

To determine the agreement between each of the four observers, the average of the measurement collected on round one and two was determined an ICC was calculated.

Technical Error of Measurement (TEM) is the preferred analysis method in anthropometry [33, 34]. Absolute TEM and relative TEM analysis was completed in Microsoft Excel (Version 2606) [35]. Previous studies which have utilised 3D models for measuring bones have set an acceptable rTEM of up to 5 [36]. However, we argue that a smaller margin be used to demonstrate accuracy. Therefore, an acceptable threshold of 2% was set for all intra- and inter-observer assessments.

The TEM is displayed as millimetres (mm). As there is not set threshold for TEM, an acceptable TEM of < 2 mm was set, given the smaller nature of the features of the mandible. The intra-observer relative TEM was calculated for the two measurement rounds for each observer.

### Inter-modality agreements

The measurements for each landmark acquired on CT, SLS, and photogrammetry were compared to determine the agreement between modalities. To achieve this, only the measurements from the second round of observation from first observer were used. Each of the nine measurements were analysed separately.

To determine intra- and inter-observer error, a TEM analysis was conducted to evaluate standard deviation and reliability between measurements. To evaluate the difference in measurements acquired, TEM analysis was also conducted with an acceptable threshold of < 5 rTEM ([36, 37].

## Results

### Intra-observer agreement

Observation rounds 1 and 2 were compared for each observer. The ICC intra-observer assessment for the four external assessors returned mostly excellent results. Refer to Table 3 for a summary of the results. Observer 1’s results were examined separately as they measured the entire sample. Observer 2 achieved results within the excellent ICC range, ranging from 0.88 to 1. Observer 3 achieved mostly good results; however, some inconsistency was detected in the measurements collected on the right ramus height, which yielded score of 0.62, although this still is classified as good. The remaining measurements from this observer were of good to excellent consistency, ranging from 0.82 to 0.94. Observer 4 also returned mostly good with some excellent, ranging from 0.84 to 0.97. Although, there was an outlier of 0.39 and 0.37 for the right ramus height and mandibular body respectively, which are poor results. Observer 5’s measurements yielded the highest consistency and accuracy, scoring between 0.94 and 0.99 for all measurements.

**Table 3:** ICC results for external observers

Measurment	O2	O3	O4	O5
Mandibular body	0.99	0.92	0.37	0.94
Bigonial breadth	1	0.88	0.84	0.98
Bicondylar breadth	1	0.83	0.88	0.98
Minimum ramus breadth (R)	0.99	0.88	0.96	0.96
Minimum ramus breadth (L)	0.96	0.93	0.97	0.96
Ramus height (R)	0.91	0.62	0.39	0.98
Ramus height (L)	0.88	0.90	0.88	0.95
Mandibular length (R)	1	0.81	0.88	0.98
Mandibular length (L)	0.99	0.94	0.86	0.99

**Table 4:** Relative and absolute TEM for each trait for intra observations of external observers. Absolute TEM is presented in parentheses

Measurement	O2	O3	O4	O5
Mandibular body height	0.79 (0.34)	1.98 (0.88)	2.23 (0.94)	1.09 (0.50)
Bicondylar breadth	0.26 (0.47)	0.88 (1.59)	0.60 (1.05)	0.26 (0.46)
Bigonial breadth	0.32 (0.48)	0.87 (1.30)	0.76 (1.07)	0.33 (0.48)
Right ramus breadth	0.93 (0.41)	1.87 (0.82)	1.42 (0.62)	1.08 (0.47)
Right ramus height	2.17 (1.95)	1.72 (1.72)	1.39 (1.30)	0.44 (0.40)
Right mandibular length	0.49 (0.65)	1.27 (1.78)	1.21 (1.59)	0.35 (0.47)
Left ramus breadth	1.56 (0.69)	1.64 (0.71)	1.09 (0.46)	1.01 (0.44)
Left ramus height	2.43 (2.24)	2.31 (2.22)	1.06 (0.93)	0.48 (0.45)
Left mandibular length	0.59 (0.79)	0.79 (1.11)	0.89 (1.18)	0. 34 (0.46)

For the intra-relative TEM results, 89% (*n* = 32/36) of measurements fell within the acceptable TEM range for this study (< 2), indicating that the observers were consistent in selecting the points for measurements across both observation rounds. 11% (*n* = 4/36) of the measurements fell outside of the acceptable TEM range, which is likely due to human error.

The relative TEM results presented in Table 4 were averaged to provide an overview of the relative TEM achieved by each external observer. Ranked from lowest to highest the average TEM of the external observers were 0.60 (Observer 5), 1.06 (Observer 2), 1.18 (Observer 4), and 1.48 (Observer 3). Observer 5 had the lowest TEM most frequently and all measurements were within the acceptable TEM range. Observer 3 had the highest TEM most frequently, scoring the highest TEM across 66.67% (*n* = 6/9) of measurements. However, 77.78% (*n* = 7/9) of Observer 3’s measurements were within the acceptable TEM threshold. Observer 2 also achieved an acceptable TEM range for 77.78% of measurements. Observer 3 had the highest overall TEM for the mandibular body height measurement; yet, all other measurements (*n* = 8/9) were within the acceptable TEM threshold.

The averages of each rTEM across observers was deduced. The analysis of these results indicate trends including that overall, bigonial breadth had the lowest TEM (0.32), followed by bicondylar breadth (0.43), mandibular body height (0.79), right mandibular length (0.65), left mandibular length (0.83), left ramus breadth (1.01), right ramus breadth (1.33), right ramus height (1.43), and left ramus height (1.57). These results highlight a strong consistency in point picking between the external observers.

Observer 1’s results are summarised in Table 5. For CT, the measurements were of moderate to excellent confidence, ranging from 0.77 to 0.94. Photogrammetry also had moderate to excellent confidence; however the range was from good at 0.60, with the higher score at 0.97. SLS had the highest confidence score overall, again with moderate to excellent results, ranging from 0.63 to 0.97. These results indicate that there was some variation in the selection of landmarks between observation rounds. However, most of the ICC results fell within the excellent range.

**Table 5:** ICC for Observer 1’s measurements across each modality using the entire sample (*n* = 33)

Measurements	CT	PG	SLS
Mandibular body	0.77	0.86	0.96
Bigonial breadth	0.70	0.72	0.63
Bicondylar breadth	0.94	0.88	0.88
Minimum ramus breadth (R)	0.92	0.95	0.74
Minimum ramus breadth (L)	0.94	0.97	0.97
Ramus height (R)	0.79	0.92	0.96
Ramus height (L)	0.82	0.94	0.93
Mandibular length (R)	0.82	0.72	0.89
Mandibular length (L)	0.79	0.60	0.84

Observer 1’s measurements from rounds 1 and 2 of observation were divided into each measurement type across each modality, using the entire sample population (*n* = 33). The TEM are represented in Table 6. For CT and photogrammetry, Observer 1 achieved an acceptable TEM range for 88.89% (*n* = 8/9) of measurements, with the CT mandibular body height measurement being outside of the acceptable TEM range (2.07), and the photogrammetry bigonial breadth measurement (2.15). All SLS measurements were within the acceptable TEM range.

**Table 6:** Observer 1 TEM results across each modality using the entire sample (*n* = 30)

Measurement type	CT	PG	SLS
Mandibular body height	2.07 (1.92)	1.52 (1.41)	1.44 (1.34)
Bicondylar breadth	0.57 (1.98)	0.75 (2.67)	0.77 (2.64)
Bigonial breadth	1.65 (4.67)	2.15 (6.16)	0.80 (2.25)
Minimum ramus breadth (R)	1.25 (1.14)	1.02 (0.91)	0.93 (0.83)
Minimum ramus breadth (L)	1.14 (1.02)	0.82 (0.72)	0.89 (0.79)
Minimum ramus height (R)	1.71 (3.19)	1.12 (2.10)	0.89 (0.79)
Maximum ramus height (L)	1.45 (2.73)	0.95 (1.77)	1.32 (2.46)
Mandibular length (R)	0.88 (2.19)	1.39 (3.84)	1.02 (2.77)
Mandibular length (L)	0.96 (2.57)	1.63 (4.50)	1.17 (3.22)

### Inter-observer agreements

All inter-observer measurements were outside of the acceptable TEM (< 2) range for this study. This indicates that further clarity and refinement is required to define the landmarks for application in a digital setting. It is important to note here that the purpose of the study is to determine if these landmarks can be measured interchangeably across modalities. Some variation may also be due to slight differences in the display, such as not zooming in or having all essential features within the frame, for example; not being able to clearly visualise both aspects of the condyles for the bicondylar distance.

Both technical TEM and ICC were calculated for each measurement type. The threshold for ICC was set at > 95 to ensure accuracy and consistency across measurements collected. The highest consistency was achieved on the left and right minimum ramus breadths, yielding a score of 0.94 and 0.93 respectively. The bigonial breadth returned an acceptable score of 70, as did the right mandibular length with 0.62. However, the mandibular body height (0.27), bicondylar breadth (0.46), ramus height (left (0.12) and right (0.34) and left mandibular length (0.50) achieved unreliable scores. All TEM measurements were above the acceptable threshold of. The results can be found in Table 7.

**Table 7:** Inter-observer TEM and ICC

Measurment	TEM	ICC
Mandibular body	3.20 (1.42)	0.27
Bigonial breadth	3.07 (5.47)	0.70
Bicondylar breadth	2.96 (4.29)	0.46
Minimum ramus breadth (R)	3.13 (1.36)	0.93
Minimum ramus breadth (L)	4.15 (3.85)	0.94
Ramus height (R)	3.51 (4.71)	0.34
Ramus height (L)	2.93 (1.26)	0.12
Mandibular length (R)	3.87 (3.55)	0.62
Mandibular length (L)	3.60 (4.82)	0.50

### Inter-modality agreement

Separate comparisons of photogrammetry and SLS measurements against CT yield ICC results that highlight distinct trends. The accuracy is excellent for both photogrammetry and SLS for mandibular body type, at 0.88 and 0.87 respectively. There was also a high consistency for measurements achieved on the rami heights, with photogrammetry returning an ICC of 0.74 and 0.82 (right and left respectively), and SLS achieving 0.84 for both. Rami breadths were also highly consistent, particularly the left ramus breadth at 0.88 for photogrammetry and 0.90 for SLS. The right ramus breadth achieved 0.77 for photogrammetry and 0.62, which reflects good accuracy between measurements. A good level of accuracy was also found for photogrammetry bigonial breadth, however a fair score of 0.47 for SLS was returned. For bicondylar breadth, photogrammetry had a fair score of 0.54, while SLS achieved 0.71. All mandibular length measurements were within an unreliable range. The photogrammetry TEM results were all within the acceptable threshold, except for right minimum ramus breadth (2.17) and left mandibular length (2.16). Similarly, SLS achieved reliable TEM results for all measurements, excluding bigonial breadth (2.37) and minimum right ramus breadth (2.59). These results are represented in Tables 8 and 9.

**Table 8:** ICC results for Observer 1 comparison between measurements achieved across modalities

Measurement type	CT v PG	CT v SLS
Mandibular body height	0.88	0.87
Bicondylar breadth	0.54	0.71
Bigonial breadth	0.70	0.47
Minimum ramus breadth (R)	0.77	0.62
Minimum ramus breadth (L)	0.88	0.90
Maximum ramus height (R)	0.74	0.84
Maximum ramus height (L)	0.82	0.84
Mandibular length (R)	0.37	0.49
Mandibular length (L)	0.29	0.50

**Table 9:** TEM score Observer 1 comparison between measurements achieved across modalities

Measurement type	CT v PG	CT v SLS
Mandibular body height	1.48 (1.36)	1.59 (1.46)
Bicondylar breadth	1.52 (5.35)	1.33 (4.64)
Bigonial breadth	1.67 (4.75)	2.37 (6.75)
Minimum ramus breadth (R)	2.17 (1.97)	2.59 (2.36)
Minimum ramus breadth (L)	1.70 (1.52)	1.53 (1.37)
Minimum ramus height (R)	1.91 (3.57)	1.55 (2.87)
Maximum ramus height (L)	1.53 (2.87)	1.58 (2.85)
Mandibular length (R)	1.81 (5.01)	1.50 (4.09)
Mandibular length (L)	2.16 (5.91)	1.95 (5.30)

**Fig. 5:**
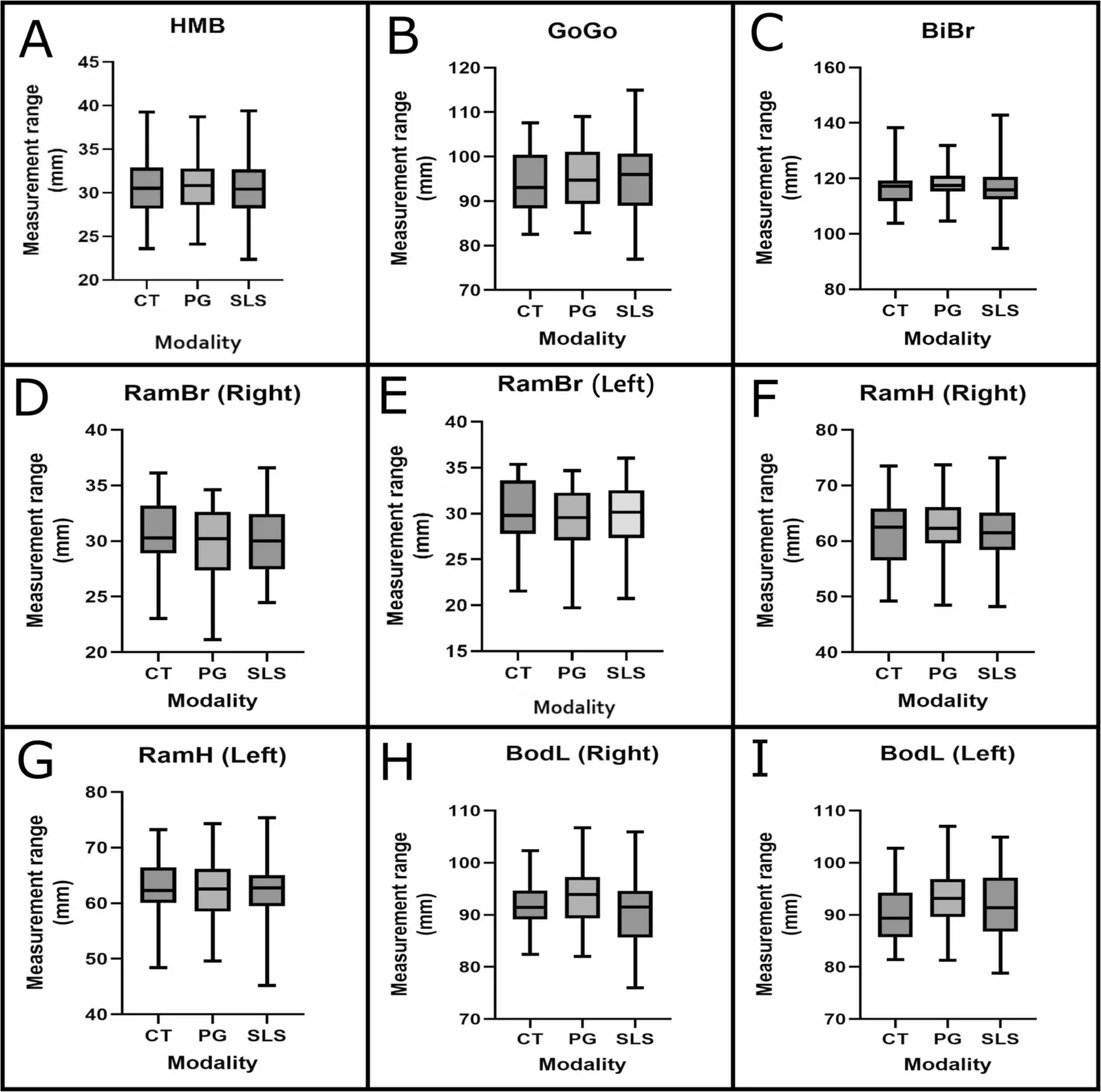
Box and whisker plots of measurement agreement per trait between modalities. Each plot represents individual traits and shows the level of consistency between each of the modalities, with the highest to lowest measurement boundaries achieved with each modality. Each panel presets data for the traits assessed in the following order: Panel **A**) HMB = Height of Mandibular Body; Panel **B**) GoGo = Bigonial Breadth; Panel **C**) BiBr = Bigonial Breadth; Panel **D** and **E**) RamBr = Ramus Breadth; Panel **F** and **G**) RamH = Ramus Height; Panel **H** and **I**) BodL = Mandibular Body Length

To provide an understanding of the distribution and variation of measurements, box plots were created using GraphPad (San Diego) and the multipanel graphic was created using Inkscape (see Fig. 5, panels A-I).

## Discussion

To date, there has been no systematic comparison of the applicability of CT, SLS, and photogrammetry within the context of metric anthropological analysis. As such, there is a scarcity of data which compares these technologies to each other. This paper answered three research questions. The first question focused on if the measurements designed for physical bone could be applied to virtual 3D models of bone. Of the twelve mandibular measurements (accounting for measurements repeated on the rami) recommended by the DCP, nine were able to be achieved using AS 20. It was not possible to achieve the gonial angle measurement as, currently, an angle tool is not available using AS 20. As the purpose of this research was to determine the accuracy of measurements across modalities and their interchangeability, the gonial angle measurement was not intrinsic. The authors intend to conduct a similar study on a software that will allow angles to be measured. The mandibular breadth also could not be measured as the AS 20 measuring tool cannot penetrate through the bone, as would be necessary to obtain this measurement.

The results indicate strong agreement between individual observer’s measuring rounds, and between modalities, although there was a lack of consistency between observers. There was also an excellent degree of consistency for measuring the rami breadth. Although images were provided to the observers to demonstrate where to place the points for measurement, the results indicate that, for most measurements, the points selected by observers varied. This may also be due to a lack of experience in measuring the bones using a 3D software, as the most experienced observer noted that zooming in close to the bone allowed a more exact selection of points. One factor that impacted the scores was that Observer 2 consistently over-measured one bone included in the subset. Observer 2 stated that they encountered a number of issues with measuring this bone in terms of centring the model and selecting the locations for measurements, making the model move around the display screen. These are issues that are commonly encountered when the model has not been correctly scaled. Instructions to avoid this occurring were provided in the study guidelines, such as selecting the box icon in the top right-hand corner and using the rough positioning feature to set the model in place prior to scaling. Therefore, the over measuring may have occurred as a result of a potential miscalculation when scaling.

Observers 2, 4, and 5 strictly followed the guidelines provided to complete the measurements, however, Observer 3 stated that they approached some measurements in an alternative manner to what was instructed. Observer 3 had the highest overall TEM for multiple measurements including right and left rami breadths, with 2.77 and 2.46 respectively, and right mandibular length at 1.89, as well as bicondylar and bigonial breadth – however, both TEM scores were within the acceptable range for a skilled observer at 1.32 and 1.31 respectively. Observer 3’s measurements for maximum ramus height were significantly different to the results of the other observers, often overscoring by more than 5 mm. Therefore, it is evident that Observer 3 misinterpreted the guidelines set for this measurement type.

These results provide insight into the application of the DCP measurements to 3D models of mandibles and the consistency of each observer in selecting the points for measurements across observation rounds and between observers. Observers 2 and 5 are actively working with physical human bone specimens in their careers. Therefore, there may be the potential that exposure to the physical human bone may be beneficial to translating measurements in the digital space. Overall, the intra-observer method indicates that the DCP measurements designed for gross bone can be replicated in the virtual space if the protocol is strictly followed.

The intra-TEM results fell mostly within the good to excellent range, with a few outliers. These results indicate that the observers were consistent in selecting their measurement points across both rounds of measuring. However, there was some variation in the inter-observer analysis. All TEM results were above the acceptable threshold for this study (< 2 TEM). Despite all observers being experienced in anatomy, it is evident from these results that there was a lack of consistency in selecting the relevant landmarks for the measurements. This may be due to misinterpreting the guidelines set out in the DCP, as discussed above. Alternatively, this may also be due to the lack of tactility that is achieved when working with physical bone that cannot be replicated with virtual 3D models. Furthermore, this may also highlight that either the DCP should be updated to include virtual analysis, including the planes and aspects required to ensure that the observers are selecting the same points, or an entirely new set of guidelines that can provide this level of detail should be created. However, significant further research is required before such guidelines could be developed. As for the ICC, only the rami breadths were within the excellent margin. Therefore, clearer definitions of the measurements need to be made to enhance the repeatability of these measurements.

The intra- and inter-observer results indicate that experience with using 3D software is not necessarily intrinsic to being able to correctly select the points for measurement. Observer 5 had the least amount of experience with 3D software but received acceptable TEM scores for most of the measurements obtained. Observer 4 also had limited experience with 3D software and achieved the lowest TEM scores overall. However, at the time of the observation, Observer 4 was actively learning and engaging with using AS. These results indicate that AS, particularly AS 20, is user-friendly and easy to learn. Observer 2 shared that they encountered some difficulties with using AS 20 for the first time, such as that the user must unselect or remove models in the objects pane to avoid the models overlapping as this resulted in the measurements being corrupted and the Observer needing to repeat the measuring. Conversely, Observer 2 stated that once this issue had been solved, AS 20 was easy to use and that they would recommend the software to other practitioners.

The second question focused on whether photogrammetry or SLS could achieve the same or similar accuracy of measurements when compared to CT. From these results, generally there was a high degree of consistency across the measurements for both photogrammetry and SLS when compared to CT. These results are important to forensic anthropology, as they successfully indicate that these modalities can be used interchangeably. To the authors’ knowledge, this is the first paper that compares the use of three 3D modalities and proves that they can be used interchangeably.

The inter-modality assessments reveal that, overall, the comparison of photogrammetry and CT yielded the highest consistency. This group also had a higher range of ICC values. The TEM analysis also returned similar results with the photogrammetry and SLS dataset having the most consistency and accuracy across the measurements. These results are interesting, since, in terms of quality, the photogrammetry models did not have as much detail as SLS and CT. However, these results are consistent with previous studies that have applied photogrammetry to other bones [26, 38]. Interestingly, there was a consistent pattern of inconsistency on longer distances. For example, ICC results for the mandibular lengths (both right and left) for both comparison types (CT-Photogrammetry and CT-SLS) were within the poor classification (ICC = < 0.50). This is likely due to human error in selecting the landmarks rather than an issue with the technology itself. However, these results raise an interesting consideration for the development of future algorithms and software that may permit automatic landmark detection and measurement workflows. Such a development will be beneficial to reduce subjectivity of identifying the relevant landmarks and points for measurements. However, further research is required to develop such tools.

### Limitations and future research

Some limitations of this study should be noted. The first of which being the small sample size of 33. The bones selected for this project were also in relatively good condition. It may be beneficial for future research to utilise a collection were bones are fragmented, as this will reflect bones that are more often encountered in forensic scenarios [36]. Furthermore, an additional limitation is that this sample could not be measured physically using a mandibulometer. Collecting the ground-truth measurements on the physical bones would have helped to demonstrate the accuracy of each of these technologies for replicating the bone. Future research should seek to compare the physical and virtual measurements to verify the accuracy of the technologies.

There were also some limitations in applying the measurements specified in the DCP. Specifically, the gonial angle and the breadth of the mandibular body could not be measured using AS 20, which limits its ability to be applicable and the applicability of the standard measurements in a digital space. Future research should seek to investigate the use of technologies that are able to facilitate these measurements by being able to measure angles and penetrate 3D models. This research would be beneficial to forensic anthropology as it will contribute to ongoing research regarding which measurements are applicable in a digital environment.

In terms of the technologies, a custom-built photogrammetry system was used, which may have contributed to the increased accuracy of photogrammetry in this instance. Future studies should compare the use of a regular DSLR camera against CT and SLS.

A significant accessibility challenge encountered throughout this study was access to laptops or PCs that had sufficient technical specifications to support the download, operation, and function of AS 20. Observer 2 also expressed a similar sentiment, in that they encountered difficulty in being able to access a computer that would allow the software to run. Initially, three other forensic anthropologists were contacted about taking part in this study, however, they all had to decline due to not being able to access a computer with enough GB and RAM to operate AS 20. This factor must be considered prior to investing in 3D technologies, particularly for research institutions.

## Conclusion

This research demonstrates how integrating advanced imaging technologies enhances the accuracy and efficiency of human identification protocols in forensic anthropology [40]. The purpose of this paper was to identify if photogrammetry and SLS could produce the same or similar accuracy to CT in terms of measurements. Photogrammetry and SLS were selected as they are more accessible, especially in terms of cost and portability, and are overall more user friendly than CT. It important to have alternative ways of documenting 3D evidence by using technologies that mitigate the limitations of CT, especially with multiple ongoing wars where the need for rapid documentation and identification is imperative. The results of this study indicate that while the modalities are interchangeable, photogrammetry yielded the highest consistency when compared to CT. These results are important, as the purpose of this study was to identify a cost-effective, lightweight, and user-friendly imaging option that could be utilised in scenarios were CT is inaccessible. Of the options explored, photogrammetry is the most cost effective, with options to utilise mobile phones successfully and apps being developed to support photogrammetry. While photogrammetry does require more training than SLS, the results are highly valuable. While SLS performed slightly less consistently than photogrammetry, the results still demonstrate a high degree of consistency between measurements. Overall, these results indicate that the decision to employ an AST, if at all, is dependent on the operator of the equipment and the individual/s assessing the 3D models.

The authors of this paper recommend that the definitions for measurements be refined so that they can be clearly applicable to virtual models to help increase repeatability. As stated by Braun et al., (2024) [39] continuing research in this area may help to establish best practice manuals. This study highlights the importance of utilising 3D technologies for documentation and analysis. Further, access to a mandibulometer has also been noted as an issue for measuring mandibles [30]. As per the DCP, a mandibulometer is required for ramus height, breadth, and length as per. However, high costs and limited access to this tool may present challenges to researchers and assessors. This challenge was encountered in this project, as the intention was to collect measurements from the physical mandible using a mandibulometer, however, a mandibulometer was unable to be sourced due to funding constraints as this tool can cost up to AUD $4,800. There is also a lack of clear instructions as to how to appropriately use a mandibulometer, which may result in incorrect measurements [40]. Therefore, an alternative approach was sought. Although virtual bone models should not replace physical bone models for training, having access to both kinds of repositories will help to ensure that practitioners are well-equipped in both types of analysis [36]. To help achieve this, future research should utilise a larger sample size and alternative bones, including post-cranial. The authors of this study also intend to apply non-metric mandibular analysis across the modality types explored in this paper.

The purpose of this paper is not to suggest that CT be replaced, but rather to demonstrate that both photogrammetry and SLS are viable alternatives, especially in scenarios where CT cannot be accessed. Rather, this study has demonstrated that all three methods are interchangeable for the purpose of measuring mandibles. This study provides a strategic framework for forensic practitioners selecting appropriate digitisation technologies. Furthermore, these findings establish a foundation for integrating digital models into existing procedures, such as the DCP, and seek to ensure that digital analysis is held to the same rigorous standard as traditional physical measurements.

## Data Availability

The data from this study cannot be openly shared but may be available upon request from the Forensic Institute Zurich. Requests to access the data should be made to the Forensic Institute Zurich.

## Footnotes

1 The perspective distortion on photogrammetry models can be reduced by setting to orthogonial view.
